# Bortezomib in multiple myeloma and lymphoma: a systematic review and clinical practice guideline

**DOI:** 10.3747/co.v13i5.106

**Published:** 2006-10

**Authors:** D. Reece, K. Imrie, A. Stevens, C.A. Smith, for the Hematology Disease Site Groupof Cancer Care Ontario’s Program in Evidence-based Care

**Keywords:** Bortezomib, Velcade, multiple myeloma, lymphoma, clinical practice guideline, systematic review

## Abstract

**Questions:**

1. In patients with multiple myeloma, Waldenström macroglobulinemia, or lymphoma, what is the efficacy of bortezomib alone or in combination as measured by survival, quality of life, disease control (for example, time to progression), response duration, or response rate?
2. What is the toxicity associated with the use of bortezomib?
3. Which patients are more or less likely to benefit from treatment with bortezomib?

**Perspectives:**

Evidence was selected and reviewed by two members of the Hematology Disease Site Group and by methodologists from the Program in Evidence-based Care (pebc) at Cancer Care Ontario. The practice guideline report was reviewed and approved by the Hematology Disease Site Group, which comprises hematologists, medical and radiation oncologists, and a patient representative. As part of an external review process, the report was disseminated to practitioners throughout Ontario to obtain their feedback.

**Outcomes:**

Outcomes of interest were overall survival, quality of life, response rates and duration, and rates of adverse events.

**Methodology:**

A systematic search was conducted of the medline, embase, HealthStar, cinahl, and Cochrane Library databases for primary articles and practice guidelines. The resulting evidence informed the development of clinical practice recommendations. Those recommendations were appraised by a sample of practitioners in Ontario and modified in response to the feedback received. The systematic review and modified recommendations were approved by a review body w theithin pebc.

**Results:**

The literature review found one randomized controlled trial (rct)—the only published rct of bortezomib in relapsed myeloma. A number of phase ii studies were also retrieved, including a randomized phase ii study. No randomized trials were retrieved for lymphoma.

The rct found bortezomib to be superior to high-dose dexamethasone for median time to progression and 1-year survival in patients with relapsed myeloma, although grade 3 adverse events were more common in the bortezomib arm. Bortezomib is recommended as the preferred treatment option in patients with myeloma relapsing within 1 year of the conclusion of initial treatment; it may also be a reasonable option in patients relapsing at least 1 year after autologous stem-cell transplantation.

**Practice Guideline:**

This evidence-based series applies to adult patients with myeloma, Waldenström macroglobulinemia, or lymphoma of any type, stage, histology, or performance status.

**Recommendations:**

Based on the results of a large well-conducted rct, which represents the only published randomized study in relapsed myeloma, the Hematology Disease Site Group (dsg) offers the following recommendations:

- For patients with myeloma refractory to or relapsing within 1 year of the conclusion of initial or subsequent treatment or treatments, including autologous stem-cell transplantation, and who are candidates for further chemotherapy, bortezomib is recommended as the preferred treatment option.
- Bortezomib is also a reasonable option for patients relapsing at least 1 year after autologous stem-cell transplantation. The dsg is aware that thalidomide, alkylating agents, or repeat transplantation may also be options for these patients. However, evaluation of these other options is beyond the scope of this practice guideline.
- For patients with myeloma relapsing at least 1 year after the conclusion of alkylating agent–based chemotherapy who are candidates for further chemotherapy, further treatment with alkylating agent–based chemotherapy is recommended.
- Evidence is insufficient to support the use of bortezomib in patients with non-Hodgkin lymphoma or Waldenström macroglobulinemia outside of clinical trials.

**Qualifying Statements:**

Limited evidence supports the appropriateness of a specific time-to-relapse period as being indicative of treatment-insensitive disease. The 1-year threshold provided in the foregoing recommendations is based on the opinion of the Hematology dsg.

For specific details related to the administration of bortezomib therapy, the dsg suggests that clinicians refer to the protocols used in major trials. Some of those details are provided here for informational purposes.

**Dosage**

Bortezomib 1.3,g/m^2^ is given as a rapid intravenous bolus over 3–5 seconds on days 1, 4, 8, and 11 of a 21-day cycle; a minimum of 72 hours between doses is required to allow for recovery of normal proteasome function. Vital signs should be checked before and after each dose. A complete blood count is recommended before each dose, with blood chemistries (including electrolyte and creatinine levels) monitored at a minimum on days 1 and 8 of each cycle. The dose of bortezomib should be reduced or held immediately upon development of painful neuropathy, as described in the product monograph; dose modification may also be required for peripheral sensory neuropathy without pain or for other toxicities. Most toxicities are reversible if dose modification guidelines are followed.

**Response to Treatment**

Responses are usually apparent by 6 weeks (2 cycles). For patients achieving complete remission (determined by negative electrophoresis and immunofixation), bortezomib should be given for 2 additional cycles beyond the date of confirmed complete remission. In patients with progressive disease after 2 cycles or stable disease after 4 cycles, dexamethasone added to the bortezomib regimen (20 mg by mouth the day of and the day after each bortezomib dose) may produce an objective response. Bortezomib (with or without dexamethasone) should be continued in patients showing benefit from therapy (excluding those in complete remission) unless disease progression or significant toxicity is observed. Therapy should be discontinued in patients who do not respond to bortezomib alone if disease progression is seen within 2 cycles of the addition of dexamethasone.

The Hematology dsg recognizes that thalidomide is an active agent in multiple myeloma patients who have relapsed after autologous stem-cell transplantation or who are refractory to alkylating agent–based chemotherapy. To date, no reported rcts have evaluated thalidomide in this role, and specifically, no trials have compared thalidomide with bortezomib. Given these limitations, the members of the Hematology dsg regard thalidomide or bortezomib as therapy alternatives to dexamethasone.

## 1. QUESTIONS

1. In patients with multiple myeloma, Waldenström macroglobulinemia, or lymphoma, what is the efficacy of bortezomib alone or in combination as measured by survival, quality of life, disease control [for example, time to progression (ttp)], response duration, or response rate?
2. What is the toxicity associated with the use of bortezomib?
3. Which patients are more or less likely to benefit from treatment with bortezomib?

## 2. CHOICE OF TOPIC AND RATIONALE

Multiple myeloma is characterized by a malignant proliferation of clonal plasma cells in the bone marrow; these cells typically produce a monoclonal immunoglobulin molecule that can be detected in serum or urine. Common manifestations include fatigue, anemia, and bone damage related to osteopenia or lytic bone lesions, or both. The bone pain, pathologic fractures, or in some cases, spinal cord compression that result from the bone damage lead to substantial morbidity. Renal failure, frequent infections, and hypercalcemia also occur in a significant proportion of patients.

Treatment of myeloma can reduce the levels of monoclonal immunoglobulins and can lead to symptomatic benefit and to delay or improvement in end-organ complications. Practice Guideline Report No. 6–6^1^ from the Program in Evidence-based Care (pebc) of Cancer Care Ontario summarizes the role of chemotherapy and stem-cell transplantation in myeloma.

Patients 65–70 years of age and younger are generally treated with several cycles of high-dose dexamethasone-based induction therapy such as vad (vincristine, doxorubicin, and dexamethasone) followed by stem-cell collection and autologous stem-cell transplantation (asct). In this patient group, asct represents the current standard of care. It has demonstrated higher remission rates (including about 20%–30% complete remissions) and better progression-free survival (pfs) and overall survival rates than has conventional chemotherapy alone.

Older patients generally receive less-aggressive therapy with oral regimens such as melphalan and prednisone. Partial remissions are seen in approximately 50% of cases, but complete remissions are rare.

Whatever the treatment approach, virtually all myeloma patients eventually relapse and require further therapy. Options for the management of recurrent myeloma include reinstitution of the initial treatment (if the original response duration was prolonged), alternative alkylating agent therapy with oral cyclophosphamide plus prednisone, high-dose dexamethasone, or thalidomide alone or in combination with corticosteroids. Therapeutic options become progressively limited as the disease progresses. At the present time, the disease is not considered curable, and overall survival rates average 3–5 years.

Over the last few years, a better understanding of the biology of myeloma cells and of the relationship between the tumour cells and the bone marrow microenvironment has stimulated efforts to develop other novel agents in this disease. Bortezomib [Velcade (Millennium Pharmaceuticals, Cambridge, MA, U.S.A.), PS-341], a first-in-class proteasome inhibitor, is the best studied of the next generation of anti-myeloma drugs.

Bortezomib blocks the action of the 29S proteasome, a multicatalytic enzyme that has been nicknamed the “housekeeper” of the cell because of its role in degrading abnormal or misfolded proteins targeted for destruction, particularly those involved in cell cycling and gene transcription. Clinical evidence suggesting that bortezomib is active in myeloma and lymphoma has begun to emerge. For this reason, the Hematology Disease Site Group (dsg) determined that, to guide appropriate use of this agent, a systematic review assessing the currently available evidence was a high priority.

## 3. METHODS

### 3.1 Guideline Development

The present systematic review is a convenient and up-to-date source of the best available evidence on bortezomib in multiple myeloma and lymphoma. The review was developed by the pebc. Evidence was selected and reviewed by two members of the pebc’s Hematology dsg. The body of evidence in the review primarily comprises data from a randomized controlled trial (rct). That evidence forms the basis of a clinical practice guideline developed by the Hematology dsg. The systematic review and companion practice guideline are intended to promote evidence-based practice in Ontario, Canada. The pebc is editorially independent of Cancer Care Ontario and the Ontario Ministry of Health and Long-Term Care.

### 3.2 Literature Search Strategy

A search of medline (Ovid, 1966 through October 2004), medline Daily Update (October 22, 2004), medline in-process and other non-indexed citations (October 22, 2004), HealthStar (1975 through September 2004), cinahl (1982 through October 2004), embase (Ovid, 1982 through 2004, week 42), and the Cochrane Library (Issue 4, 2004) databases was conducted. Literature searches were not restricted for publication type or study design.

In addition, conference proceedings of the American Society of Clinical Oncology (1995–2004) and the American Society of Hematology (1996–2004) were searched for abstracts of relevant trials. The Canadian Medical Association Infobase (mdm.ca/cpgsnew/cpgs/index.asp), the National Guidelines Clearinghouse (www.guideline.gov/index.asp), and the National Institute for Clinical Excellence (www.nice.org.uk/) were also searched for existing evidence-based practice guidelines.

Relevant articles and abstracts were selected and reviewed by two reviewers, and the reference lists from these sources were searched for additional trials. Personal files were also searched.

### 3.3 Study Selection Criteria

Articles of study designs of any type (including systematic reviews, meta-analyses, and evidence-based practice guidelines) were selected for inclusion in this systematic review of the evidence if they were fully published reports or published meeting abstracts in the English language, and if they were

- studies that included adult patients with myeloma, Waldenström macroglobulinemia, or lymphoma (any histologic subtype, stage, performance status, or disease type).
- studies evaluating bortezomib as a single agent or in combination with other regimens.
- comparative trials of bortezomib (the bortezomib could be compared with any agent, any combination of agents, or placebo).
- reports of one or more of the following outcomes: survival, quality of life, disease control (for example, ttp), response duration, response rate, or adverse effects.

Studies were excluded if they were

- letters, comments, books, notes, or editorials.
- studies reporting fewer than 20 patients (all disease types combined).

### 3.4 Synthesizing the Evidence

Because of the small sample of rcts retrieved, the Hematology dsg decided not to pool the results obtained. The primary outcome of interest was pfs; secondary outcomes of interest were response rate and overall survival. Subset analyses were conducted according to histology.

## 4. RESULTS

### 4.1 Literature Search Results

A total of 344 database citations and conference proceedings were evaluated in the original literature search. Agreement between the two reviewers who scored the database and conference publications for inclusion was κ = 0.84. After retrieval, thirteen studies were excluded:

- One did not report information separately for the patient populations of interest.
- One did not meet the sample size criterion.
- Seven were previous reports of included trials.
- Four were abstracts reporting combined data from included trials.

From the original and updated literature searches, twenty publications of sixteen trials were located. Two trials were located by searching personal files. Tables I, II, and III provide an overview of the sixteen trials.

Of the sixteen trials meeting the inclusion criteria, eleven deal with myeloma, and five deal with lymphoma. No reports were located for Waldenström macroglobulinemia.

For myeloma (Table I), we located one rct abstract (apex, Assessment of Proteasome Inhibition for Extending Remissions study^18^—the full-report^2^ was retrieved later), one randomized phase ii trial (crest, Clinical Response and Efficacy Study of Bortezomib in the Treatment of Relapsing Multiple Myeloma)^3^, four nonrandomized phase ii trials (summit, Study of Uncontrolled Multiple Myeloma Managed with Proteasome Inhibition Therapy, in abstract and full report form^4^,^19^, and three other trial abstracts^9^,^11^,^12^), and five dose-escalation trial abstracts^5^–^8^,^10^. One additional abstract^20^ reporting toxicity data of an included trial^12^ was also located. One previous report^21^ of an included trial^7^ provided toxicity data for that trial. Of those eleven trials, seven were in relapsed or refractory myeloma, and four were in previously untreated patients.

For lymphoma, we located four nonrandomized phase ii trials (one abstract and full report of the same trial^13^,^22^, and three other abstracts^14^–^16^) and one nonrandomized phase i/ii trial^17^. Relapsed or refractory patients were evaluated in three trials^15^,^17^,^22^, and a mix of previously treated and untreated patients were evaluated in two trials^13^,^14^,^16^.

### 4.2 Outcomes

#### 4.2.1 Question 1

In patients with multiple myeloma, Waldenström macroglobulinemia, or lymphoma, what is the efficacy of bortezomib alone or in combination as measured by survival, quality of life, disease control (for example, ttp), response duration, or response rate?

##### Multiple Myeloma

###### Survival

Survival data were reported in five studies: the rct, the randomized phase ii trial, and three nonrandomized studies. The rct^2^, which compared bortezomib with dexamethasone, reported a 14% greater 1-year survival in the bortezomib arm (Table II), with a hazard ratio of 0.57 (*p* = 0.001). In other trials, median survival ranged from 16 to 26.7 months^3^,^4^,^7^, with the longest survival being seen in the crest trial^3^ (performed in less extensively pretreated patients).

###### Quality of Life

No published analysis compares bortezomib to other agents with regard to quality of life. In the phase ii nonrandomized summit trial, quality-of-life data were reported to have improved in incomplete and partial responders to bortezomib, but not in minimal or non-responders^9^.

###### Disease Control

Four trials reported data on ttp^2^–^5^. The median ttp reported in two phase ii trials (one randomized and one nonrandomized) ranged from 7 to 11 months. In the rct, median ttp was significantly longer with bortezomib than with dexamethasone (6.2 months vs. 3.5 months, *p* < 0.001).

Event-free survival (efs) data were reported in one dose-escalation trial^7^. The median efs for combined bortezomib and thalidomide was 7 months. The 12-month efs was 20% for the cohort receiving bortezomib 1.0 mg/m^2^ plus daily thalidomide 150 mg, and 26% for the cohort receiving bortezomib 1.0 mg/m^2^ plus daily thalidomide 200 mg.

Response duration was reported in the rct^2^, being 8 months with bortezomib as compared with 5.6 months with dexamethasone (*p* = unreported). The summit and crest trials^3^,^4^ also reported response duration, with values ranging from 9.5 months to 13.7 months.

###### Response

Table II summarizes the overall response rates to bortezomib alone or in combination with other agents, according to disease status. Reporting varied among the trials: Some trials reported only complete and partial responses; others included minimal responses. In the rct, the response rate to bortezomib was significantly greater than that to dexamethasone (38% vs. 18%, *p* < 0.001). Responses to bortezomib as a single agent ranged from 33% to 96%. Response rates reported for bortezomib in combination with other agents ranged from 18% to 84%.

##### Lymphoma

Table III shows data for the use of bortezomib in malignant lymphoma. All trials evaluated single-agent bortezomib, and one trial included an arm combining bortezomib and chemotherapy. Response rates ranged from 7% to 55%.

#### 4.2.2 Question 2

What is the toxicity associated with the use of bortezomib?

##### Multiple Myeloma

In eight trials^2^–^6^,^10^–^12^, a variety of grade 3 or 4 adverse events in varying frequencies were reported, including neutropenia (eight trials); thrombocytopenia (five trials); neuropathy (five trials); diarrhea or fatigue (three trials each); abdominal pain, anemia, dyspnea, hyponatremia, pneumonia with no other symptoms, pneumonia, sepsis, and vomiting (two trials each); and anorexia, bone pain, constipation, cough, deep vein thrombosis (dvt), dizziness, fever, headache, insomnia, lymphopenia, mucositis, nausea, non-neutropenic infection, orthostatic hypotension, pain in limb, paresthesia, pyrexia, rash, syncope, or weakness (one trial each).

Grade 3 neuropathy was reported in four trials^2^–^4^,^11^, and grade 4 neuropathy in two of those^2^,^3^. One trial explicitly stated that no grade 3 or 4 neuropathy was observed^6^.

In the rct^2^, a higher proportion of patients in the bortezomib arm than in the dexamethasone arm had one or more grade 3 adverse events (*p* < 0.01). Patients experienced one or more of the following grade 3 adverse events: anorexia, diarrhea, neuropathy, neutropenia, and thrombocytopenia. Grade 4 adverse events were more common in the bortezomib arm than in the dexamethasone arm for thrombocytopenia (4% vs. 1%, *p* = 0.05) and neutropenia (2% vs. 0%, *p* = 0.01). Of 21 reported adverse events of any grade, 17 occurred in a statistically significantly greater proportion of patients in the bortezomib arm than in the dexamethasone arm. One additional study^12^ reported toxicity data, but did not state whether any toxicity reached grade 3 or 4. One abstract^20^ that included dvt data for two trials reported 0% dvt occurrence in a trial evaluating bortezomib combination therapy before transplant^12^. Toxicity was not reported in one trial^8^. The earlier report^21^ of Zangari *et al.* 2004^7^ provided the toxicity data for that trial.

Discontinuation of treatment because of toxicity was reported in four trials^2^–^4^,^11^, with 5% to 37% of patients discontinuing treatment. Discontinuation because of bortezomib treatment–related toxicity in the rct was 37% (as compared with 29% for the dexamethasone arm), and in the phase ii trials^3^,^4^, discontinuation ranged from 15% to 18%. A subset of patients in three trials discontinued treatment because of neuropathy^2^–^4^. Another trial^11^ reported one grade 3 neuropathy event leading to discontinuation.

The rct^2^ reported 4 possible bortezomib treatment-related deaths (3 from cardiac causes, 1 from sudden death of unknown cause). The summit trial^4^ reported 2 possible treatment-related deaths, and the Barlogie *et al.* trial^12^ reported 1 treatment-related death in a patient with renal failure at baseline. The crest trial^3^ reported 1 death attributable to pneumonia in a patient receiving bortezomib at 1.3 mg/m^2^. One trial^6^ explicitly stated that no toxicities were fatal.

##### Lymphoma

Most of trials in lymphoma evaluated bortezomib monotherapy in relapsed or refractory patients. Five trials reported toxicities. In the full report by O’Connor *et al.*^13^, the grade 3 toxicities observed were lymphopenia (14 patients); thrombocytopenia (7 patients); hypokalemia, hyponatremia, infection without neutropenia, neuropathy, and prolonged pro-thrombin time (2 patients each); and alanine aminotransferase, alkaline phosphatase, hemoglobin, hyperkalemia, leukocytes, nausea, neutrophils, anorexia, constipation, and fatigue (1 patient each). Only 1 grade 4 event was observed: hyponatremia. The abstract update of that trial reported similar data.

Among three trials published in abstract form^14^,^15^,^17^, similar grade 3 and 4 toxicities were reported (thrombocytopenia, gastrointestinal side effects, neuropathy, fatigue, anemia, and neutropenia). The fourth trial^16^, also in abstract form, evaluated bortezomib in previously treated and untreated patients and reported grade 2 or higher toxicities attributed to the study drug. These toxicities were similar to those mentioned earlier (anorexia, gastrointestinal side effects, fatigue, dizziness, sensory neuropathy, edema, hypotension, vascular leak syndrome, arthralgia, myalgia, neuropathic pain, dyspnea, and rash).

In the trial^17^ that evaluated bortezomib plus ep-och chemotherapy (etoposide, vincristine, doxorubicin, cyclophosphamide, prednisone), grade 4 neutropenia, grade 4 thrombocytopenia, fever and neutropenia (grade not given), grade 2 or higher gastrointestinal toxicities, and grade 3 or higher sensory neurotoxicity were observed. The authors of that trial also compared the toxicities in the combination-therapy arm with those in a historical cohort of patients receiving fixed-dose epoch. Results were similar for fever and neutropenia (grade not given), grade 4 neutropenia, and grade 3 and 4 thrombocytopenia, but were higher in the fixed-dose epoch group for grade 2 or higher gastrointestinal toxicity (statistical analysis not provided). Grade 2 or higher neurotoxicity was also more frequent with fixed-dose epoch, but the authors stated that fewer treatment cycles were received by the combination-therapy patients.

In the full report by O’Connor *et al.*^13^, 2 patients were taken off the study because of toxicity, and 13 patients (50%) missed at least one dose of bortezomib. Thrombocytopenia was the most common reason for missed bortezomib doses, and it occurred most frequently at the beginning of the study before the investigators changed the platelet count requirements (≥ 100,000 to ≥50,000/μL for the first dose of every cycle). Thrombocytopenia was the only dose-limiting hematologic toxicity.

In the trial by Goy *et al.*^14^, 1 patient with *Herpes zoster* died of encephalitis. In the trial by Belch *et al.*^16^, toxicity led to discontinuation in 9 patients, including 6 because of neuropathy or myalgia. Amendment of eligibility criteria to exclude patients with edema, dyspnea, or effusion at baseline eliminated the occurrence of serious toxicities.

#### 4.2.3 Question 3

Which patients are more or less likely to benefit from treatment with bortezomib?

##### Multiple Myeloma

In the summit trial, the factors reported to predict for higher response rates, complete (cr) or partial (pr), with bortezomib monotherapy werean age less than 65 years and bone-marrow plasmacytosis of 50% or less (*p* < 0.05 by multivariate analysis)^4^. Known adverse prognostic factors such as β_2_-microglobulin level, number of prior therapies, and chromosome 13 abnormalities were not found to predict for response. In the abstract update of that trial, a partial least-squares regression analysis detected that high serum protein, bone-marrow plasma cells, and β_2_-microglobulin, and low platelet count, serum albumin, hemoglobin, Karnofsky score, body surface area, weight, and quality of life at baseline predicted for mortality^19^.

In the apex trial^2^, the authors stratified the randomization using prognostic factors such as number of prior therapies and then conducted subgroup analyses. Time to progression, 1-year survival, response rate (cr and pr), and duration of response were higher in patients with one prior line of therapy than in those with more than one prior line of therapy, although the study design did not permit statistical comparisons of the effect of prognostic factors.

One trial^7^ evaluating bortezomib combination therapy in refractory myeloma analyzed 17 factors by multivariate analysis and concluded that treatment more than 5 years earlier was associated with superior survival (*p* = 0.03), previous thalidomide treatment was associated with inferior survival after combination treatment (*p* = 0.05), and bortezomib at the 1.3 mg/m^2^ dose reduced the risk of death (*p* = 0.02).

##### Lymphoma

Although the numbers of patients treated were small, and no statistical analyses have been performed to date, the highest response rates to bortezomib have been observed in mantle cell and follicular lymphoma.

## 5. DISCUSSION

The standard first-line therapy for myeloma was established through a series of large randomized trials. That topic is addressed in the pebc Practice Guideline No. 6–6, “Optimal Therapy for Patients Diagnosed with Multiple Myeloma and the Role of High-Dose Chemotherapy and Stem Cell Support”^1^. For patients with advanced-stage myeloma and good performance status, asct is recommended as first-line therapy. For patients not eligible for asct, oral alkylating agent–based chemotherapy with regimens such as melphalan, or prednisone and cyclophosphamide, represent the standard of care. Neither approach is curative, but both are associated with moderate-to-high rates of remission, effective palliation of symptoms, and acceptable toxicity profiles.

Bortezomib has not been compared with standard treatment options in rcts of first-line treatments. Until evidence of its superiority to currently available treatment options becomes available, the Hematology dsg does not recommend that bortezomib be used as first-line therapy outside the setting of a clinical trial.

The optimal therapy for patients beyond first-line therapy is not well established. Available options include additional alkylating agent–based chemotherapy or regimens of high-dose dexamethasone, thalidomide, or (more recently) bortezomib. Of these options, only bortezomib has been tested in rcts involving relapsed patients.

Alkylating agents (for example, melphalan, cyclophosphamide, prednisone) as second-line therapy may produce successful re-induction in cases of relapsed myeloma. Where relapse occurs following the use of alkylating agents as first-line therapy, additional therapy with alkylating agents may effectively palliate symptoms and induce remissions, while offering the advantage of convenient oral administration and a relatively favourable toxicity profile^23^. Fewer data are available on the use of alkylating agents for relapse following asct or high-dose therapy. A reasonable expectation is that toxicity, particularly myelosuppression, would be greater in this setting, given the limited marrow reserve following transplantation.

High-dose oral dexamethasone alone has modest activity in relapsed and refractory myeloma, and it is an appropriate comparator for tests of new agents or regimens either as a single agent or in combination with vincristine and adriamycin in the vad regimen^24^,^25^. Because of a lack of myelosuppression, this regimen is commonly used in patients with compromised bone marrow reserves. Although vad has not been compared to high-dose dexamethasone alone in randomized trials, it is generally believed to be modestly more effective—but at the cost of increased toxicity and inconvenience. The vad regimen must be administered intravenously through a central venous catheter and is associated with myelosuppression, alopecia, nausea, and peripheral neuropathy.

Thalidomide is also an active agent in relapsed and refractory myeloma^26^. Data from uncontrolled trials show a response rate of 30%–40%, with a proportion of patients remaining disease-free for a prolonged period. The addition of corticosteroids appears to improve the response rates^27^.

Thalidomide is administered orally and is not associated with myelosuppression, but it does have significant toxicities, particularly neurotoxicity, that may cause discontinuation of therapy in some patients. Thalidomide is also highly teratogenic. Thalidomide has not been compared with other agents in randomized trials in relapsed or refractory patients. It is approved for use by the U.S. Food and Drug Administration under a special program to safeguard against birth defects. However, it has not been approved by the Canadian Health Protection Branch and is therefore not widely available in Canada.

In patients with relapsed myeloma, the dsg emphasized the importance of sensitivity to alkylating agents in defining the optimal therapeutic regimen. Patients who remain sensitive to alkylating agents may be effectively re-treated with alkylators. No consensus definition for alkylator sensitivity exists, but the dsg thought that the commonly used relapse threshold of 1 year or more after alkylating agent–based chemotherapy was a reasonable definition. No other regimen has been compared to re-treatment with alkylating agents in this group of patients.

In view of the favourable toxicity profile and greater ease of administration of alkylating agent–based regimens, the dsg recommends treatment or re-treatment with alkylating agent chemotherapy for patients with relapsed myeloma whose disease is sensitive to alkylating agents, including patients whose first-line treatment was asct or high-dose therapy and who are candidates for further chemotherapy. The oral regimen of weekly cyclophosphamide (250–300 mg/m^2^, usually 500 mg) and every-second-day prednisone (50–100 mg) is commonly used and produces less cumulative myelosuppression than does the combination of melphalan and prednisone^28^. Thalidomide-based therapy is also an option under these circumstances.

For patients with myeloma refractory to first-line treatment (that is, with relapse occurring within 1 year of treatment) who are candidates for chemotherapy, the use or reuse of alkylating agents is not a reasonable option. Treatment options under these circumstances include high-dose dexamethasone, thalidomide, and bortezomib. When compared with high-dose dexamethasone in the apex trial, bortezomib was associated with a 14% improvement in 1-year survival without increased severe toxicity. The dsg considers this difference to be important. Although thalidomide has demonstrated activity in this population of patients in uncontrolled studies, it has not been compared to either bortezomib or dexamethasone. For this reason, the dsg considers that bortezomib is the preferred treatment option for this group of patients.

The place of bortezomib in the management of lymphoma and Waldenström macroglobulinemia was also considered. Only preliminary results from small studies are available for these cancers. Until such time as more mature data are available, the dsg does not recommend that bortezomib be used in patients with these diagnoses outside of clinical trials.

## 6. EXTERNAL REVIEW OF THE PRACTICE GUIDELINE REPORT

This systematic review and practice guideline was distributed for review and feedback to practitioners throughout Ontario, Canada, in accordance with the Practice Guidelines Development Cycle^29^,^30^.

### 6.1 Methods

The practice guideline recommendations were submitted with the systematic review to a sample of 161 hematologists, medical oncologists, and radiation oncologists in Ontario. The survey consisted of items evaluating the methods, results, and discussion used to inform the draft recommendations and asking whether the draft recommendations should be approved as a practice guideline. Written comments were invited. The practitioner feedback survey was mailed on November 15, 2005, and reminder cards and complete repeat mailings were sent in the following weeks. The Hematology dsg reviewed the results of the survey.

### 6.2 Results of the External Review

The response rate for the survey was 78 of 161 questionnaires mailed (48%). Of the 78 respondents, 46 (59%) indicated that they cared for patients for whom the guideline is relevant and completed the survey.

Overall, the respondents showed strong support for the guideline. For questions that addressed issues such as the rationale for the guideline, the quality of the guideline, and the clarity of the recommendations, a substantial majority of respondents (93%–100%) expressed modest-to-strong support (score of 1 or 2 on a scale of 1–5: 1 = “strongly agree,” 3 = “neither agree or disagree,” 5 = “strongly disagree”) for the report.

With respect to the appropriateness of the recommendations, an overwhelming majority of respondents, 41–43 of 46 (89%–93%) agreed with the draft recommendations and their appropriateness for the specified target population. A strong majority (78%) also felt that the recommendations were not excessively rigid and could be applied to individual patients.

A strong majority responded positively to all but 4 of the 23 questions. The items with lower rates of positive responses were related to the feasibility of implementing the recommendations or to economic issues. When asked about the need to reorganize practice to accommodate these guidelines, 37% of respondents felt that there would be a need to reorganize practice, 20% were ambivalent, and 43% did not feel that there would be such a need. Similarly, when asked if they felt that implementing the draft recommendations would be technically challenging, 21% agreed, 37% were ambivalent, and 42% disagreed.

With respect to costs, only 33% of respondents disagreed with the statement that the “recommendations are too expensive to apply,” with 35% being ambivalent, and 33% feeling that implementation was economically feasible. When asked if they felt that the recommendations would result in practice that would be more resource-efficient, only 30% of respondents agreed; a significant proportion responded with ambivalence (43%) or disagreed (11%).

#### 6.2.1 Written Comments

1. One respondent felt that there should be separate reports for bortezomib in myeloma and for bortezomib in other diseases.
2. Two respondents felt that the scope of the recommendations should be expanded and that bortezomib should be recommended for use in patients with mantle cell lymphoma.
3. One respondent felt that the clause “outside of clinical trial” should be added to the recommendations for non-Hodgkin lymphoma or Waldenström macroglobulinemia.
4. One respondent acknowledged that there was no direct comparison between thalidomide and bortezomib and indicated uncertainty about when to recommend one agent over the other.
5. One respondent felt that the qualifying statements should set out a specific duration of treatment for patients who respond to bortezomib.
6. One respondent stated that it was not clear that re-treatment is more effective than bortezomib in “alkylator-sensitive” patients.
7. One respondent stated that the use of bortezomib after front-line melphalan, prednisone, or thalidomide may specifically lead to increased toxicity.
8. One respondent thought that the observed effectiveness of bortezomib may be a result of the limited availability of the drug for use in patients.
9. One respondent felt the that the discussion should address the discrepancy in the apex study between the reported improvement in pfs at less than 3 months and the 14% improvement at 1 year.
10. Six respondents felt that the funding status for bortezomib was a major issue for this guideline. Some felt that the guideline should be approved only if bortezomib receives funding.

#### 6.2.2 Modifications/Actions

The dsg reviewed and addressed the written feedback as follows:

1. The systematic review of the literature was designed to retrieve studies of bortezomib in patients with myeloma, Waldenström macroglobulinemia, or lymphoma because the dsg was aware that this agent had been studied in each of these diseases. Given the similarity in dosing of this agent for each indication, presenting the data in a single document was felt to be appropriate, with the data presented separately for each indication. The dsg will update the present guideline report as new evidence becomes available; updates may include the development of separate reports for specific disease populations.
2. The dsg is aware of emerging evidence regarding the efficacy of bortezomib in the mantle cell lymphoma population. At the present time, the dsg feels that the evidence is insufficient to support the use of bortezomib in these patients.
3. The dsg agreed that the addition of this clause would make the recommendations clearer.
4. The systematic review did not identify any trials that directly compare bortezomib with thalidomide in the patient population under consideration. In the absence of such data, the dsg is unable to provide more specific advice on the relative role of the two agents.
5. Qualifying statements pertaining to the treatment regimen are not intended to be recommendations for practice; they are instead intended to serve an informational purpose. The dose administration data and other treatment information provided are taken from the protocols used in the major trials evaluated for this report. The dsg will determine if additional information can be provided on the duration of treatment for bortezomib.
6. The dsg acknowledges that no direct comparison of bortezomib with re-treatment using alkylating agent–based therapy has been published. In the absence of such data, the dsg favours the use of alkylator re-treatment in patients known to be sensitive to alkylating agents, because this treatment is effective, non-toxic, more convenient, and much less expensive.
7. The systematic review evaluated the toxicity associated with bortezomib use in patients who had received prior therapy. The dsg felt that the toxicity rates were acceptable.
8. It is generally true that patients enrolled in clinical trials may differ in some important respects from the overall patient population (for example, they may be healthier, or they may represent the most severe cases) and that this discrepancy may bias outcomes. Nonetheless, the dsg felt that the principal rct informing these recommendations (the apex trial) was of appropriate quality and sufficiently generalizable to the target patient population.
9. In the apex trial, the bortezomib arm showed a statistically significant improvement in survival and pfs. The dsg considered the 14% improvement in survival to be clinically important. The dsg cannot comment on the perception of a difference in magnitude of the improvement at the two endpoints.
10. Four respondents stated that bortezomib was too costly. One stated that the costs outweighed the benefits, and that the toxicity may outweigh benefits as well. The pebc guidelines are designed primarily to address clinical concerns, including outcome measures related to efficacy and toxicity. Economic analyses of the impact of agents are not included in this assessment.

The dsg notes that, generally speaking, feedback for this report was positive for questions related to the report development process and was supportive of the recommendation for bortezomib use (for example, in patients with multiple myeloma who relapse within 1 year of treatment). The main areas of reviewer disagreement were related to the funding and implementation of bortezomib in Ontario. Some respondents felt that implementing the recommendations in this report would be technically challenging and that the cost of the drug coupled with the fact that it is not currently funded in the province, would be a barrier to its widespread use in Ontario.

In light of feedback provided by external reviewers, the DSG made the following modifications to the report:

- The clause “(including autologous stem-cell transplantation)” was added to the first recommendation regarding bortezomib use.
- The clause “outside of clinical trial” was added to the recommendations for non-Hodgkin lymphoma or Waldenström macroglobulinemia.
- Additional information on the duration of treatment for bortezomib was provided in the Qualifying Statements.

### 6.3 Report Approval Panel Feedback

The finalized evidence-based series report was reviewed and approved by the pebc Report Approval Panel in March 2006. The panel consists of two members with expertise in clinical and methodology issues (including an oncologist). No significant issues were raised by the panel, and the report was approved for distribution.

## Figures and Tables

**TABLE I:** Summary of the 12 myeloma trials meeting the inclusion criteria

*Study*	*Patient characteristics*	*Treatment*	*Evaluable [*n *(%)]*
Relapsed/refractory myeloma — phase iii rcts			
Richardson *et al.* 2004^2^ (apex)	Relapsed mm (1–3 prior regimens); dexamethasone-refractory disease excluded	Bortezomib 1.3 mg/m^2^ vs. Dexamethasone 40 mg	315 (95%) 312 (93%)
Relapsed/refractory myeloma — phase ii extension study trials			
Jagannath *et al.* 2004^3^ (crest)	mm in relapse or refractory to first-line therapy, including asct	Bortezomib 1.0 mg/m^2^ *Bortezomib + dexamethasone 20 mg* vs. Bortezomib 1.3 mg/m^2^ *Bortezomib + dexamethasone 20 mg*	27 (96%) *16* 26 (100%) *12*
Richardson *et al.* 2003^4^ (summit)	Relapsed mm and refractory to salvage chemotherapy	Bortezomib 1.3 mg/m^2^ *Bortezomib + dexamethasone 20 mg*	193 (96%) *74*
Relapsed/refractory myeloma — combination therapy and dose escalation			
Berenson *et al.* 2004^5^ (abstract)	Previously treated refractory/relapsed mm	Bortezomib 0.7 mg/m^2^^a^, plus melphalan 0.025–0.25 mg/kg dose cohorts	24 (92%)
Hollmig *et al.* 2004^6^ (abstract)	High-risk, advanced mm	Bortezomib 1.0 or 1.3 mg/m^2^, plus melphalan 100–250 mg/m^2^, plus asct	27 (73%)
Zangari *et al.* 2004^7^ (abstract)	Refractory mm after transplant and salvage treatment^b^	Bortezomib 1.0 mg/m^2^^c^, plus daily thalidomide 50–200 mg dose cohorts^d^	79
Orlowski 2003^8^ (abstract)	Refractory mm	Bortezomib 0.9–1.5 mg/m^2^, plus pegylated doxorubicin 30 mg/m^2^	22 (92%)
Newly diagnosed myeloma — phase ii monotherapy			
Richardson *et al.* 2004^9^ (abstract)	Previously untreated, symptomatic mm	Bortezomib 1.3 mg/m^2^	22 (79%)
Newly diagnosed myeloma — combination therapy and dose escalation			
Wang *et al.* 2004^10^ (abstract)	Newly diagnosed mm	Bortezomib 1.0–1.9 mg/m^2^, plus thalidomide 100–200 mg daily, plus dexamethasone 20 mg/m^2^	25 (100%)
Newly diagnosed myeloma — combination therapy before asct			
Jagannath *et al.* 2004^11^ (abstract)	Newly diagnosed mm patients; asct at physician discretion	Bortezomib 1.3 mg/m^2^ *Bortezomib + dexamethasone 20 mg*	23 (61%) *14*
Barlogie *et al.* 2004^12^ (abstract)	Newly diagnosed mm	Bortezomib, thalidomide, dexamethasone, plus pace, plus peripheral blood stem cell collection, melphalan-based asct	57 (100%)

a In the absence of dose-limiting toxicity, bortezomib increased to 1.0 mg/m^2^.

b One patient did not receive a transplant as per a previous report of this trial^10^.

c Bortezomib increased to 1.3 mg/m^2^ in absence of grade 3 neurotoxicity.

d To-date accrual done to bortezomib 1.3 mg plus thalidomide 150 mg.

rct = randomized controlled trial; apex = Assessment of Proteasome Inhibition for Extending Remissions study; mm = multiple myeloma; crest = Clinical Response and Efficacy Study (of Bortezomib in the Treatment of Relapsing Multiple Myeloma); asct = autologous stem-cell transplantation; summit = Study of Uncontrolled Multiple Myeloma Managed with Proteasome Inhibition Therapy; pace = 4-day infusion of cisplatin 10 mg/m^2^, doxorubicin 10 mg/m^2^, cyclophosphamide 400 mg/m^2^, and etoposide 40 mg/m^2^.

**TABLE II:** Trials evaluating bortezomib regimens in multiple myeloma

*Trial*	*Treatment*	*orr*^a^*(%)*	*cr(%)*	*pr(%)*	*Medianttp(months)*	*Overall survival*^b^
Relapsed/refractory myeloma — phase iii rcts						
Richardson *et al.* 2004^2^ (apex)	Bortezomib (1.3 mg/m^2^)	38	6	32^c^	6.2	80% at 1 year
	Dexamethasone (40 mg)	18	1	17^c^	3.5	66% at 1 year
Relapsed/refractory myeloma — phase ii extension study trials						
Jagannath *et al.* 2004^3^ (crest)	Bortezomib 1.0 mg/m^2^ daily	33	4	26^c^	nr	nr
	*Bortezomib + dexamethasone*	*44*	*7*	*30*^c^	*7*^d^	*26.7 months*^d^,^e^
	Bortezomib 1.3 mg/m^2^	50	4	35	nr	nr
	*Bortezomib + dexamethasone*	*62*	*4*	*46*	*11*^d^	*Not reached*^d^,^e^
Richardson *et al.* 2003^4^ (summit)	Bortezomib 1.3 mg/m^2^	35	4	24^c^	7^f^	16 months^d^
	*Bortezomib + dexamethasone*	*18*	*0*	nr	*6.6*	nr
Relapsed/refractory myeloma — combination therapy and dose escalation						
Berenson *et al.* 2004^5^ (abstract)	Bortezomib 0.7 mg/m^2^^g^, plus melphalan dose cohorts	67	4	29 c,h,i	1–18	nr
Hollmig *et al.* 2004^6^ (abstract)	Bortezomib 1.0 or 1.3 mg/m^2^, plus melphalan, plus asct	39	26	13	nr	nr
Zangari 2004^7^ (abstract)	Bortezomib 1.0 mg/m^2^^j^, plus thalidomide dose cohorts^k^	60^h^	0	~60^c^,^l^	7 (efs)	21 months
Orlowski 2003^8^ (abstract)	Bortezomib 0.9–1.5 mg/m^2^, plus pegylated doxorubicin	68	23	45^c^	nr	nr
Newly diagnosed myeloma — phase ii monotherapy						
Richardson *et al.* 2004^9^ (abstract)	Bortezomib 1.3 mg/m^2^	64	5	36	nr	nr
Newly diagnosed myeloma — combination therapy and dose escalation						
Wang 2004^10^ (abstract)	Bortezomib (1.0–1.9 mg/m^2^), plus thalidomide, plus dexamethasone	84	na	84	nr	100% at 6 months
Newly diagnosed myeloma — combination therapy before asct						
Jagannath 2004^11^ (abstract)	Bortezomib 1.3 mg/m^2^ *Bortezomib + dexamethasone*	96	13 *0*	70^c^ m	nr	nr
Barlogie *et al.* 2004^12^ (abstract)	Bortezomib, thalidomide, dexamethasone, plus pace, plus peripheral blood stem cell collection, melphalan-based asct	26^n^	26^n^	0	nr	nr

a Sum of all measured response rates (for example, complete response rate + near-complete response rate + partial response rate + minimal response rate).

b Percentage values indicate percentage of patients surviving; time values indicate median patient survival time.

c Partial response rate and near-complete response rate.

d Patients continuing in the extension study known or assumed to be included in these analyses.

e Data assumed to be for patients receiving bortezomib ± dexamethasone.

f Discrepancy within article concerning whether 202 or 196 patients were included in the analysis.

g Bortezomib increased to 1.0 mg/m^2^ in the absence of dose-limiting toxicity.

h Complete response rate and near-complete response rate occurred in bortezomib 1.0 mg/kg + melphalan 0.025 mg/kg cohort.

i Partial response or better observed in those with prior bortezomib or melphalan treatment.

j Bortezomib increased to 1.3 mg/m^2^ in the absence of grade 3 neurotoxicity.

k One patient not evaluable for response.

l A small number of nodular complete remissions (~20) included in this value.

m 64% improved.

n 40% after first transplant.

orr = overall response rate; cr = complete response rate; pr = partial response rate; ttp = time-to-progression; rct = randomized controlled trial; apex = Assessment of Proteasome Inhibition for Extending Remissions study; nr = not reported; crest = Clinical Response and Efficacy Study (of Bortezomib in the Treatment of Relapsing Multiple Myeloma); summit = Study of Uncontrolled Multiple Myeloma Managed with Proteasome Inhibition Therapy; asct = autologous stem-cell transplantation; efs = event-free survival; pace = 4-day infusion of cisplatin 10 mg/m^2^, doxorubicin 10 mg/m^2^, cyclophosphamide 400 mg/m^2^, and etoposide 40 mg/m^2^.

**Table III:** Trials evaluating bortezomib therapy in malignant lymphoma

*Trial*	*Treatment and patients*	n^a^	*orr*^b^*(%)*	*cr(%)*	*pr(%)*	*Median remission (months)*
Phase ii, nonrandomized						
O’Connor 2004^13^ (multicentre)	Bortezomib 1.5 mg/m^2^ Relapsed, refractory, or untreated indolent nhl and mcl	51 (100%)^c^	55	nr	nr	nr
		*fl: 19*	*60*	*5*	*5*^d^	*Not yet reached*
		*mcl: 23*	*56*	nr	nr	*6–19*
		*sll/cll: 5*	*20*	*0*	*20*	nr
		*mzl: 4*	*100*	*0*	*100*	nr
Goy 2002^14^ (abstract)	Bortezomib 1.5 mg/m^2^ Relapsed/refractory lymphoma; median age 63 years; median of 4 prior treatments; entry: ≤ grade 1 sensory neuropathy	24/30 (80%)	38	13	25	nr
		*sll: 1*	*0*			
		*fl: 2*	*0*			
		*mcl: 15/18*	*53*	*20*	*33*	
		*dlbcl: 6/8*	*17*	*0*	*17*	*2–7*^e^
		*tfl: 0/1*	ne			
Strauss 2004^15^ (abstract)	Bortezomib 1.3 mg/m^2^ Relapsed/refractory lymphoma subset; median of 3.5 prior treatments	32/32 (100%)	19^f^	3	16	nr
		*mcl: 11*	*36*	*9*	*27*	
		*fl: 10*	*0*^g^	*Not assessable*	*Not assessable*	
		*wm: 4*	*50*	*0*	*50*	
		*ll: 1*	*0*	na	na	
		*dlbcl: 1*	*0*	na	na	
		*atl: 1*	*0*	na	na	
		*dfcl: 1*	*0*	na	na	
		*hd: 3*	*0*	na	na	
Belch 2004^16^ (abstract)	Bortezomib 1.3 mg/m^2^ Mantle cell lymphoma; advanced stage previously untreated or ≤2 prior chemotherapy regimens; median age 67 years; stage iii/iv disease in all cases	24/30 (80%)	33^h^	0	33^h^	nr
		*pt: 14*	*36*^h^	*0*	*36*	
		*ut: 10*	*30*	*0*	*30*	
Phase i/ii						
Dunleavy 2004^17^ (abstract)	Bortezomib 1.3 mg/m^2^	With chemotherapy: 25/26 (96%)	24	8	16	nr
	Relapsed/refractory aggressive B-cell lymphoma (activated B-cell dlbcl); median age 54 years; median of 4 prior therapies	Alone: 15/16 (94%)	7	0	7	nr

a Subgroups indicated by italic font.

b Sum of all measured response rates: for example, compete response rate + near-complete response rate + partial response rate + minimal response rate.

d One patient was untreated.

d Unconfirmed complete response rate/partial response rate not reported.

e Lower end represents 1 patient with autologous stem-cell transplantation; 2–7 refers to complete remission; for partial remission, value was 3 months.

f Plus a 6% “late response” rate.

g “Late response” was recorded by authors in 2 patients (7%).

h Unconfirmed complete response rate + partial response rate.

orr = overall response rate; cr = complete response rate; pr = partial response rate; nr = not reported; nhl = non-Hodgkin lymphoma; mcl = mantle cell lymphoma; fl = follicular lymphoma; sll = small lymphocytic lymphoma; cll = chronic lymphocytic leukemia; mzl = marginal zone lymphoma; dlbcl = diffuse large B-cell lymphoma; tfl =transformed fl; ne = not evaluable; wm = Waldenström macroglobulinemia; ll = lymphoplasmacytic lymphoma; na = not applicable; atl = adult T-cell leukemia/lymphoma; dfcl = diffuse follicle centre lymphoma; hd = Hodgkin disease; pt = previously treated; ut = untreated.
